# Goodpasture's Syndrome Following Radiation Exposure: A Rare Pulmonary-Renal Complication

**DOI:** 10.7759/cureus.107499

**Published:** 2026-04-21

**Authors:** Sina Djoo, Lorie Seuylemezian, Aerin Mellott, Nahyun M Kim, Johnpaul Singh

**Affiliations:** 1 Internal Medicine, University of the Incarnate Word School of Osteopathic Medicine, San Antonio, USA; 2 Internal Medicine, Western University of Health Sciences, Pomona, USA; 3 Internal Medicine, College of Osteopathic Medicine of the Pacific, Western University of Health Sciences, Pomona, USA; 4 Psychiatry, Mission Community Hospital, Panorama City, USA; 5 Internal Medicine, Mission Community Hospital, Panorama City, USA

**Keywords:** goodpasture's syndrome, pulmonary-renal syndrome, radiation effect, rapidly progressing glomerulonephritis, respiratory support

## Abstract

Goodpasture’s syndrome (anti-glomerular basement membrane (anti-GBM) disease) is a rare, life-threatening autoimmune condition characterized by rapidly progressive glomerulonephritis and pulmonary hemorrhage. We describe a woman in her 60s with a history of breast cancer treated with lumpectomy and radiotherapy who developed hemoptysis, hematuria, acute kidney injury, and respiratory failure. She lacked traditional environmental risk factors, like cigarette smoking, hydrocarbon exposure, respiratory infections, and inhalational lung injury associated with anti-GBM disease. Diagnosis was established through positive circulating anti-GBM antibodies and a confirmatory lung biopsy demonstrating linear IgG deposition along the alveolar basement membrane. Her course was complicated by respiratory failure requiring tracheostomy, recurrent infections, anemia, and transient dialysis dependence. The patient improved following aggressive immunosuppression, plasmapheresis, and supportive care. This case highlights the diagnostic challenges of anti-GBM disease in critically ill patients, and although a causal relationship cannot be established from a single case, we evaluate how prior radiotherapy may have contributed to disease development in a susceptible individual.

## Introduction

Anti-glomerular basement membrane (anti-GBM) disease, also known as Goodpasture’s syndrome, is a rare autoimmune small-vessel vasculitis characterized by circulating antibodies directed against the α3 chain of type IV collagen in glomerular and alveolar basement membranes. The disease often presents with rapidly progressive crescentic glomerulonephritis and pulmonary hemorrhage [1,2]. It is an uncommon cause of pulmonary-renal syndrome but carries significant morbidity and mortality if not recognized early [1]. Known risk factors include smoking, cocaine use, hydrocarbon exposure, mechanical stress, and human leukocyte antigen (HLA)-associated genetic susceptibility, particularly HLA-DRB1*1501. Reports of radiotherapy as a potential trigger are rare, and radiotherapy has only rarely been discussed as a possible trigger for anti-GBM disease. One prior report described anti-GBM glomerulonephritis following radiotherapy for early prostate cancer [1]. However, evidence remains limited, and causality cannot be established from isolated reports. Here, we present a case of anti-GBM disease arising after prior breast cancer treatment with radiotherapy in a patient without traditional environmental risk factors, highlighting a rare and clinically complex pulmonary-renal presentation.

## Case presentation

A woman in her 60s with a past medical history of breast cancer treated with lumpectomy and radiotherapy, deep vein thrombosis (DVT), anemia, and hypertension was transferred from another facility for continued management of presumed anti-GBM disease, ventilatory support, and further diagnostic evaluation.

The patient initially presented to the first medical center with concern for gastrointestinal (GI) bleed, anemia, and acute kidney injury (AKI). She was admitted to the intensive care unit (ICU) for severe anemia and transfused with blood products. Esophagogastroduodenoscopy (EGD) was performed, and a gastric ulcer was found and treated with a proton pump inhibitor (PPI) and medical management. Nephrology was consulted for AKI, and renal function worsened despite medical management. After being downgraded from the ICU, she was readmitted to the ICU due to worsening respiratory distress and renal failure and was started on dialysis. She required bilevel positive airway pressure (BiPAP) and eventually a tracheostomy with ventilator support due to respiratory failure. Renal biopsy was not performed despite worsening renal failure on dialysis because of her worsening clinical status. In lieu of a renal biopsy due to unstable clinical status, anti-GBM titers were tested and found to be positive. An inferior vena cava (IVC) filter was placed after finding DVTs; the patient was not a good candidate for anticoagulation, given significant pulmonary hemorrhage with hemoptysis and GI bleeding. Dialysis continued twice per week, and rituximab treatment was completed with minimal clinical improvement.

At the time of transfer, the working diagnosis was based on her prior presentation with hemoptysis, hematuria, AKI, anemia, and respiratory failure, concerning for a pulmonary-renal syndrome. On admission, the patient remained on mechanical ventilatory support, and the initial plan focused on gradual weaning and eventual transition to subacute rehabilitation once medically stable. During the first two hospital days, nephrology followed the patient closely for renal dysfunction. Because her kidney function was relatively stable at that time, continuation of dialysis was deferred, although close monitoring was recommended, along with consideration of renal biopsy if she became stable enough to tolerate the procedure.

By hospital day 3, ongoing hemoptysis prompted a pulmonology consultation. CT of the chest demonstrated active pneumonitis, and corticosteroid therapy was resumed. Given the persistent diagnostic uncertainty and concern for ongoing pulmonary involvement, a lung biopsy was recommended if she remained hemodynamically stable.

On hospital day 11, the vascular and thoracic surgery team performed an open lung biopsy with chest tube placement. Following the procedure, the patient was transferred to the ICU for closer monitoring after failing a continuous positive airway pressure (CPAP) weaning trial. High-dose methylprednisolone was initiated, with plans for a gradual prednisone taper. On hospital day 13, the chest tube was successfully removed, and postprocedural chest radiography demonstrated a small residual pneumothorax. Pathologic examination of the lung biopsy revealed linear IgG deposition along the alveolar basement membrane, confirming the diagnosis of Goodpasture’s syndrome. Circulating anti-GBM antibodies were positive, supporting the diagnosis of anti-GBM disease [2,3]. Thus prompting initiation of treatment, she underwent extensive immunosuppressive therapy, including plasmapheresis, rituximab, intravenous immunoglobulin, and high-dose corticosteroids. Dialysis was initiated due to renal failure. On hospital day 14, endocrinology was consulted for glucose monitoring in the setting of high-dose corticosteroid therapy, and by hospital day 15, serum glucose had risen to 191 mg/dL, prompting initiation of an insulin regimen for glycemic control.

The remainder of the hospitalization was marked by several complications requiring multidisciplinary management. On hospital day 18, laboratory testing demonstrated worsening renal function, with blood urea nitrogen increasing to 170 mg/dL and creatinine to 1.6 mg/dL, prompting reinstitution of one session of hemodialysis with continued monitoring thereafter. On hospital day 20, hemoglobin declined to 6.6 g/dL, necessitating transfusion of packed red blood cells. By hospital day 21, progressive leukocytosis raised concern for superimposed infection, and infectious disease consultation was obtained. Sputum and urine cultures grew Pseudomonas aeruginosa, and antimicrobial therapy was tailored to ciprofloxacin and tobramycin based on susceptibility data.

After treatment of these complications, the patient’s clinical status gradually improved. On hospital day 24, she was downgraded from the ICU to the telemetry floor. On hospital day 28, she developed urinary retention; renal ultrasonography demonstrated a nonobstructing left renal stone, no hydronephrosis, and possible calculi within the urinary bladder, and a Foley catheter was placed. By hospital day 29, her oxygen requirement had improved from 3 L/min to 1 L/min by nasal cannula.

After hospital day 30, her hospital course was largely unremarkable. She remained clinically stable and stayed hospitalized primarily for observation and coordination of placement at an appropriate rehabilitation facility for ongoing recovery and physical strengthening. The patient was later transferred for continued stabilization, ventilator weaning, and rehabilitation under multidisciplinary care.

Investigations

**Figure 1:**
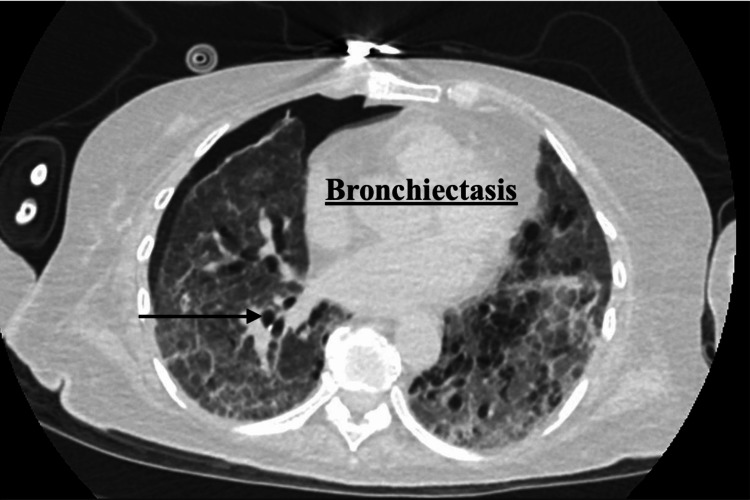
CT scan imaging: bronchiectasis Bronchiectasis is a chronic, irreversible dilation and destruction of the bronchial walls resulting from recurrent infection, inflammation, or impaired mucociliary clearance. For our patient, we hypothesize this injury to be related to radiation therapy received for her breast cancer. CT, computed tomography

**Figure 2:**
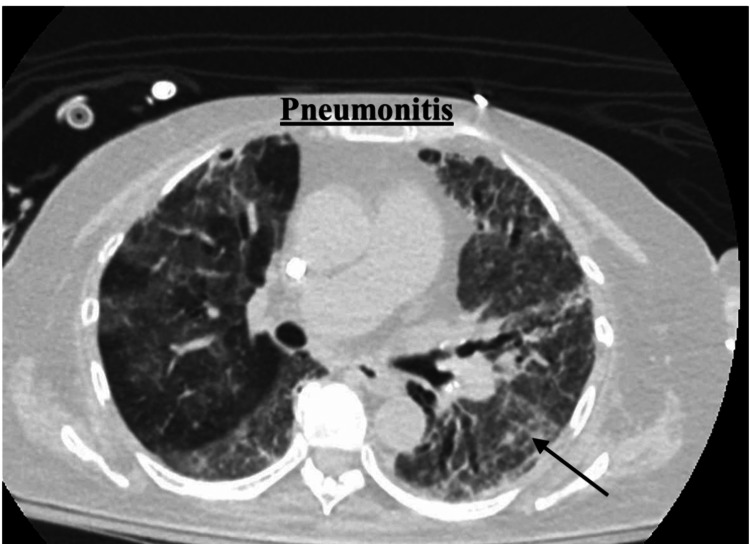
CT scan imaging: pneumonitis The pulmonary manifestation of anti-GBM disease is characterized by immune-mediated pneumonitis secondary to autoantibody-driven injury of alveolar basement membranes. These are seen as ground glass opacities in our patient and secondary to an ongoing inflammatory process, suspicious for an autoimmune cause. CT, computed tomography

**Figure 3:**
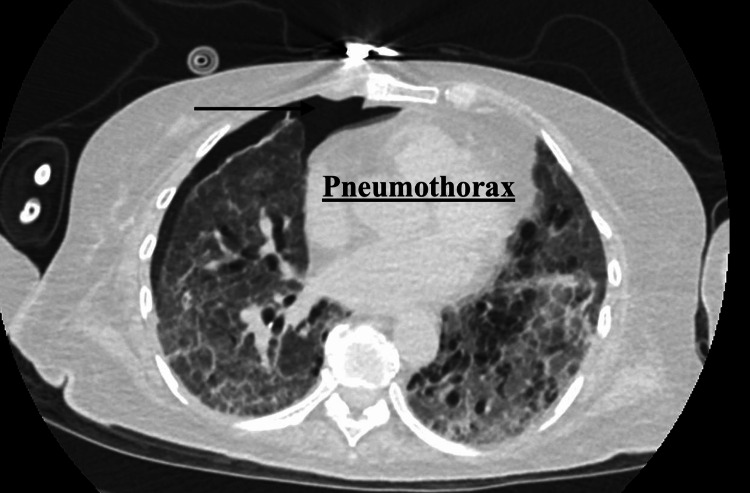
CT scan imaging: pneumothorax A pneumothorax is defined as the presence of air within the pleural space, which causes partial or complete collapse of the underlying lung. This occurs when there is a disruption of the visceral or parietal pleura due to trauma, lung disease, or spontaneous rupture of alveoli or blebs. For our patient, this was seen secondary to her open lung biopsy with chest tube placement and subsequent removal. CT, computed tomography

Serologic testing demonstrated elevated circulating anti-GBM antibodies. Computed tomography (CT) of the chest revealed pneumonitis with diffuse ground-glass opacities and bronchiectasis (Figure 1 and Figure 2). Bronchoscopy showed diffuse pulmonary hemorrhage. An open lung biopsy of the right upper and lower lobes revealed diffuse interstitial fibrosis with hemosiderin-laden macrophages and mixed inflammatory infiltrates. Immunohistochemical staining demonstrated IgG positivity along the alveolar basement membrane, supporting the diagnosis of Goodpasture’s syndrome. Acid-fast bacilli and fungal stains were negative. Following biopsy, imaging demonstrated a small pneumothorax related to chest tube removal (Figure 3).

Laboratory studies were notable for severe anemia requiring transfusion, leukocytosis during infectious complications, and elevated blood urea nitrogen and creatinine levels with transient dialysis dependence. Renal biopsy was not performed due to clinical instability. Laboratory findings on admission and peak abnormalities during hospitalization, including positive anti-GBM antibodies, are summarized in Table 1.

**Table 1:** Key laboratory findings WBC, white blood cell count; Hgb, hemoglobin; Hct, hematocrit; BUN, blood urea nitrogen; eGFR, estimated glomerular filtration rate; CRP, C-reactive protein; BNP, B-type natriuretic peptide; anti-GBM, anti-glomerular basement membrane

Test	Admission	Peak/nadir	Units	Reference range
WBC	13.9	30.7	×10³/µL	4.0-11.0
Hgb	9.7	6.6	g/dL	12-16
Hct	29.3	20	%	36-46
Platelet count	166	109	×10³/µL	150-400
BUN	90	170	mg/dL	7-20
Creatinine	1.4	1.6	mg/dL	0.6-1.2
eGFR	44	38	mL/min/1.73m²	>60
CRP	-	17.3	mg/L	<3.0
BNP	-	302	pg/mL	<100
Anti-GBM antibodies	Positive	Positive	-	Negative

Differential diagnosis 

The patient’s presentation with hemoptysis, AKI, anemia, and progressive respiratory failure raised immediate concern for a pulmonary-renal syndrome. The initial diagnostic framework centered on immune-mediated, infectious, and malignancy-associated processes capable of causing diffuse alveolar hemorrhage and rapidly progressive renal dysfunction.

Antineutrophil cytoplasmic antibody (ANCA)-associated vasculitis was a primary consideration, as granulomatosis with polyangiitis and microscopic polyangiitis frequently present with pulmonary hemorrhage and crescentic glomerulonephritis. However, the absence of upper airway involvement, lack of granulomatous inflammation on lung biopsy, and the presence of circulating anti-GBM antibodies made ANCA-associated vasculitis less likely. Additionally, lung histopathology did not demonstrate necrotizing vasculitis, which would be expected in ANCA-mediated disease.

Infection-related causes were also carefully evaluated, given the patient’s anemia, pulmonary infiltrates, and subsequent leukocytosis. Severe bacterial pneumonia, opportunistic infections, and pulmonary hemorrhage secondary to infection were considered. Bronchoscopy revealed diffuse alveolar hemorrhage without evidence of localized infection, and lung biopsy demonstrated no fungal organisms, acid-fast bacilli, or necrotizing infection. While the patient later developed secondary Pseudomonas aeruginosa infections during her prolonged hospitalization, these occurred after the onset of pulmonary hemorrhage and were therefore considered complications rather than the primary etiology.

Malignancy-associated pulmonary-renal syndromes and paraneoplastic processes were considered, particularly in the setting of prior breast cancer. However, imaging and histopathologic examination showed no evidence of recurrent or metastatic malignancy. Lung biopsy was negative for dysplasia or neoplasia, and the pattern of linear IgG deposition along the alveolar basement membrane was inconsistent with malignancy-driven pathology.

Drug-induced pulmonary hemorrhage and renal injury were also considered, including anticoagulant-related bleeding and medication-induced interstitial lung disease. Anticoagulation was contraindicated and avoided due to active bleeding, and no offending medications known to cause alveolar hemorrhage or immune-mediated nephritis were identified prior to symptom onset.

Ultimately, the diagnosis of anti-GBM disease was supported by the constellation of clinical findings, positive circulating anti-GBM antibodies, and a confirmatory lung biopsy demonstrating linear IgG deposition along the alveolar basement membrane. Although renal biopsy is the diagnostic gold standard, it was contraindicated due to hemodynamic instability. In this context, lung biopsy served as a critical diagnostic alternative, allowing exclusion of competing etiologies and confirmation of Goodpasture’s syndrome.

Treatment

Treatment consisted of aggressive immunosuppression with high-dose intravenous methylprednisolone followed by a gradual oral prednisone taper, rituximab, and intravenous immunoglobulin. The patient underwent 22 sessions of plasmapheresis to remove circulating anti-GBM antibodies. Supportive care included mechanical ventilation with tracheostomy, intermittent hemodialysis during periods of azotemia, red blood cell transfusions for anemia, and infection prophylaxis. She developed Pseudomonas aeruginosa pneumonia and a urinary tract infection, which were treated with targeted antibiotic therapy. Prophylaxis against Pneumocystis jirovecii was maintained with atovaquone.

Standard therapy for anti-GBM disease typically includes plasmapheresis together with glucocorticoids and cyclophosphamide. In this case, rituximab and intravenous immunoglobulin were selected instead of a cyclophosphamide-based regimen in the setting of prolonged critical illness, hemodynamic instability, and infectious complications, where additional cytotoxic therapy was felt to carry increased risk. This individualized approach reflected the complexity of balancing disease control against treatment tolerance in a critically ill patient.

Outcome

The patient’s respiratory status gradually improved, allowing transition to CPAP and eventual weaning to low-flow supplemental oxygen. Renal function stabilized without the need for ongoing dialysis. Hemoglobin levels improved following transfusion, and leukocytosis resolved after completion of antibiotic therapy. She was discharged to a subacute rehabilitation facility with plans for continued physical therapy, oxygen weaning, tracheostomy decannulation, and Foley catheter removal. At discharge, laboratory values included a hemoglobin level of 10 g/dL, creatinine of 1.3 mg/dL, and an estimated glomerular filtration rate of 48 mL/min/1.73 m².

## Discussion

Anti-GBM disease is a rare, life-threatening small-vessel vasculitis characterized by autoantibodies targeting the α3 chain of type IV collagen in glomerular and alveolar basement membranes, leading to rapidly progressive glomerulonephritis and, in up to 60% of cases, diffuse alveolar hemorrhage (Goodpasture’s syndrome) [1-3]. The disease often presents acutely, with high morbidity and mortality if not promptly recognized and treated [1,4].

The pathogenic autoantibodies cause direct injury to glomerular and pulmonary capillaries, resulting in crescentic glomerulonephritis and pulmonary hemorrhage [2,5]. Environmental triggers and genetic susceptibility are implicated in disease onset [3,6]. Diagnosis is based on clinical presentation, detection of circulating anti-GBM antibodies, and kidney biopsy showing linear IgG deposition along the GBM [2,3].

Our patient was suspected of having anti-GBM disease, but did not have a definitive diagnosis from her prior hospitalization. Although the gold standard for the diagnosis of anti-GBM disease is a kidney biopsy showing linear IgG deposits over the basement membrane, our patient was clinically deemed to be too hemodynamically unstable to undergo a kidney biopsy. Instead, definitive diagnosis came from a combination of clinical presentation, elevated anti-GBM antibodies, and a lung biopsy showing hemosiderin-laden macrophages with interstitial fibrosis and positive IgG staining. In such cases, an alternative tissue biopsy may provide sufficient diagnostic confirmation when renal biopsy is contraindicated [3,5,7].

In this case, we present a patient with classic features of anti-GBM disease (i.e., Goodpasture’s syndrome) yet without the usual environmental triggers (no smoking, no illicit drug use, no known hydrocarbon or metal exposure). This strengthens the plausibility of her prior radiotherapy for breast cancer as a potential precipitant. Ionizing radiation has been shown to induce sustained tissue injury, oxidative stress, and inflammatory cytokine release [8-10]. Radiation may cause cell necrosis and release of damage-associated molecular patterns (DAMPs) that activate dendritic cells and downstream T-cell responses, facilitating autoimmunity [9]. In the context of anti-GBM disease, such an insult could expose normally sequestered epitopes of the α3 chain of type IV collagen in the glomerular or alveolar basement membranes, triggering an autoreactive B-cell response [9].

Although direct reports of radiotherapy triggering anti-GBM disease are rare, the mechanistic pathway of radiation-induced immune dysregulation offers a biologically plausible explanation in patients without traditional risk factors [9,10]. One similar case has been reported in a patient who developed anti-GBM disease following radiotherapy for prostate cancer [1]. Our case highlights that in patients lacking common exposures, prior ionizing-radiation therapy could be considered as a potential trigger for anti-GBM disease in genetically susceptible individuals. At the same time, this case does not establish causality. Other unmeasured host factors, immune susceptibility, or antecedent pulmonary injury may also have contributed to disease onset. Accordingly, the most appropriate interpretation is that this case is hypothesis-generating rather than confirmatory. It expands the differential for possible anti-GBM triggers and supports additional study of whether radiation exposure may serve as one of several contributing insults in predisposed individuals.

The cornerstone of therapy is rapid initiation of immunosuppression combined with daily plasma exchange to remove circulating antibodies and suppress further production. Plasmapheresis is continued until anti-GBM titers are undetectable on two consecutive tests. Cyclophosphamide is typically administered for two to three months, and glucocorticoids are tapered over six months. Supportive care includes aggressive management of respiratory failure (e.g., ventilatory support), transfusion for anemia, and renal replacement therapy as needed. Infection prophylaxis is critical due to profound immunosuppression; prophylaxis against Pneumocystis jirovecii and monitoring for opportunistic infections are recommended. Our patient had received a tracheostomy and was eventually able to tolerate CPAP.

In summary, early recognition of disease and aggressive combined immunosuppression and plasmapheresis are essential for optimal outcomes in anti-GBM disease. Prognosis is largely dependent on the severity of renal involvement at presentation, particularly initial creatinine levels and the need for dialysis [4,8].

## Conclusions

This case highlights the importance of maintaining a high index of suspicion for anti-GBM disease in patients presenting with pulmonary-renal syndrome, even in the absence of traditional environmental risk factors. Our patient developed diffuse alveolar hemorrhage, AKI, and respiratory failure with positive anti-GBM antibodies and a confirmatory lung biopsy demonstrating linear IgG deposition along the alveolar basement membrane. Although kidney biopsy remains the diagnostic gold standard, this case illustrates that lung biopsy combined with serologic testing can establish the diagnosis when renal biopsy is contraindicated due to hemodynamic instability. Early recognition and prompt initiation of plasmapheresis and immunosuppressive therapy were critical for stabilization and recovery. Additionally, the absence of common triggers such as smoking or hydrocarbon exposure raises the possibility that prior radiotherapy may represent a potential precipitating factor for autoimmune activation in susceptible individuals. Although the temporal relationship and biologic plausibility raise the possibility that prior radiotherapy may have contributed to disease development, causality cannot be inferred from a single case. Rather, this report should be viewed as a hypothesis-generating observation that adds to the limited literature on possible radiation-associated anti-GBM disease and supports continued investigation into this potential association.
